# 慢性髓细胞性白血病中国诊断与治疗指南（2025年版）

**DOI:** 10.3760/cma.j.cn121090-20250923-00437

**Published:** 2025-12

**Authors:** 中华医学会血液学分会

## Abstract

2025年版慢性髓细胞性白血病（CML）中国诊断与治疗指南在2020年版基础上，结合近5年来CML诊疗领域进展进行修订。参照2022国际共识分类（ICC）修订分期标准，明确CML急淋变中淋巴母细胞阈值。慢性期患者预后分层推荐ELTS评分系统，同时推荐源自中国多中心数据的酪氨酸激酶抑制剂（TKI）治疗失败预测模型，兼顾遗传学、分子学异常的预后意义。治疗药物新增阿思尼布、奥雷巴替尼、泊那替尼，阐述一线及后线治疗的决策原则。里程碑反应对后续治疗策略调整具有指导意义，但强调应个体化解读分子里程碑反应。首次提出在结合疗效和安全性基础上进行TKI剂量优化；无治疗持续缓解实施标准及停药后监测、生育相关问题有所调整，旨在保证疗效基础上，最大限度减少不良反应、改善生活质量。异基因造血干细胞移植依然是多种TKI耐药/不耐受以及进展期患者的根治性手段。

## Abstract (en)

The guideline for the diagnosis and treatment of chronic myeloid leukemia（CML）in China（2025 edition）has been revised based on its 2020 version, incorporating advances in the field of CML diagnosis and treatment over the past 5 years. With reference to the revised 2022 International Consensus Criteria, the threshold for lymphoblasts in lymphoid blast transformation is clearly defined. The EUTOS Long-Term Survival score is recommended to evaluate the patient's risk status at diagnosis. Meanwhile, a prediction model for tyrosine kinase inhibitor（TKI）treatment failure derived from Chinese multicenter data is also recommended. The prognostic significance of cytogenetic and molecular abnormalities should be considered. Newly therapeutic drugs include asciminib, olverembatinib, and ponatinib. This version of the guideline systematically elaborates on the decision-making principles for first-line and later-line medications. Milestone responses are of guiding significance for treatment strategy adjustments; however, we emphasize that molecular milestone responses should be interpreted individually. Concurrently, it proposes TKI dose optimization based on efficacy and safety. The implementation criteria for treatment-free remission, postdiscontinuation monitoring, and fertility-related issues have been adjusted to minimize toxicities and improve quality of life while ensuring efficacy. Allogeneic hematopoietic stem cell transplantation remains valuable for patients with multidrug TKI resistance or intolerance and those in advanced phases.

慢性髓细胞性白血病（Chronic myeloid leukemia，CML）是骨髓造血干细胞克隆性增殖形成的恶性肿瘤，占成人白血病的15％[1]。CML全球发病率因地区不同存在一定差异。我国1986至1988年在全国22个省市、自治区46个调查点进行的全国白血病发病情况调查显示CML的年发病率为0.36/10万[2]。此后国内几个地区的流行病学调查显示CML的年发病率为（0.39～0.55）/10万[3]–[6]。欧洲EUTOS（European Treatment and Outcome Study）调查2008–2012年人群发病率为0.99/10万[7]，美国SEER（Surveillance Epidemiology and End Results）数据观察1975–2009年年标准化人群发病率为1.75/10万[8]。由于CML的发病率随着年龄的增长而升高[7]–[8]，地区间的差异在一定程度上可能是由人群年龄分布所致。中国CML患者较西方更为年轻化，国内的流行病学调查显示CML中位发病年龄为45～50岁[9]–[11]，而美国CML的中位发病年龄为66岁[12]。

传统靶向ATP（Adenosine triphosphate）位点的一代酪氨酸激酶抑制剂（Tyrosine kinase inhibitors，TKI）伊马替尼作为一线药物使CML患者的10年生存率达85％～90％[13]，尼洛替尼、达沙替尼、氟马替尼等二代TKI一线治疗CML能够获得更快更深的分子学反应，逐步成为一线治疗方案之一[14]–[17]。创新机制靶向ABL1肉豆蔻酰口袋（Specifically targeting ABL myristoyl pocket, STAMP）的变构药物阿思尼布与经典一代、二代TKI治疗新诊断CML慢性期的随机对照研究显示患者可获得更快更深的分子学反应以及更佳的耐受性，美国食品药品监督管理局（Food and Drug Administration，FDA）及中国国家药品监督管理局（National Medical Products Administration，NMPA）已批准阿思尼布用于CML慢性期一线治疗[18]。目前愈来愈多的临床研究数据表明，TKI治疗获得持续的深度分子学反应（Deep molecular response，DMR）超过2年的患者，部分能够获得长期的无治疗缓解（Treatment free remission, TFR），即功能性治愈[19]。尽快获得完全细胞遗传学反应（Complete cytogenetic response，CCyR）以及更深的分子学反应是CML治疗近期目标，改善生活质量和功能性治愈是CML治疗的长期目标，功能性治愈成为越来越多CML患者追求的治疗目标。异基因造血干细胞移植（allo-HSCT）曾经是CML的一线治疗选择，目前仅作为多种TKI耐药/不耐受患者尤其是进展期患者根治性治疗选择。随着三代TKI泊那替尼、奥雷巴替尼和STAMP变构药物阿思尼布的获批和普及，耐药之后的后线治疗选择越来越多，在CML的治疗中应该详细评估患者的全面情况后，参考患者的治疗意愿，向其推荐优势治疗选择。

根据已有的临床研究证据，结合中国的实际情况，参照2025年《慢性髓细胞性白血病NCCN肿瘤学临床实践指南》[20]（NCCN 2025）、欧洲白血病网（ELN 2013、2020、2025）专家组的治疗推荐[21]–[23]，经过国内血液学专家讨论后制订本指南，为血液科医师和肿瘤科医师提供最新的临床指导。本共识已在国际实践指南注册与透明化平台（Practice guideline REgistration for transPAREncy, PREPARE）注册（注册号：PREPARE-2025CN1771）。

一、诊断分期及预后分组

（一）诊断分期

1. 诊断标准：大多数CML患者在常规体检和血常规检测时被发现。对不明原因的白细胞增高，伴或不伴有血小板增多、血红蛋白降低或增高，尤其伴随嗜碱性或嗜酸性粒细胞增加时应当考虑CML可能。常见症状包括疲劳、脾肿大引起的腹部不适。检测到Ph染色体和（或）BCR::ABL1融合基因阳性方可诊断CML。5％～10％患者存在变异Ph染色体[24]，1％～5％患者表现为隐匿性BCR::ABL1重排［常规染色体核型分析无法检测到Ph染色体，通过荧光原位杂交（FISH）、逆转录酶聚合酶链反应（RT-PCR）检测可明确诊断］[23]，约2％患者携带罕见BCR::ABL1转录本，如e19a2、e1a2、e13a3或e14a3等[25]。初始诊断推荐通过细胞遗传学、RT-PCR明确是否存在BCR::ABL1重排，明确BCR::ABL1转录本类型，同时应进行外周血和骨髓细胞形态学检查明确疾病分期[20],[26]。

2. CML的分期：参照2022国际共识分类（ICC）修订[27]。

（1）慢性期（Chronic phase，CP）：①外周血或骨髓中原始细胞<10％；②没有达到诊断加速期或急变期的标准。

（2）加速期（Accelerate phase, AP）：符合至少1项下列指标：①骨髓或外周血原始细胞10％～19％；②外周血嗜碱性粒细胞≥20％；③初诊或治疗过程中Ph染色体阳性基础上出现下列额外的染色体异常（Additional chromosomal abnormalities, ACA）：+Ph、+8、i（17q）、+19、3q26.2重排、复杂核型。

（3）急变期（Blastic phase, BP）：符合至少1项下列指标：①骨髓或外周血原始细胞≥20％；②骨髓活检显示成片原始细胞聚集浸润；③髓外原始细胞浸润；④形态学上出现淋巴母细胞>5％时需要考虑CML急淋变（免疫分型证实原始细胞的淋系起源）。

（二）预后评估

CML分期依然是疾病结局最强的预后特征，针对慢性期有许多因素影响长期治疗结局，有多个预后评分系统，均以临床特点及血液学指标作为预后评分因素。TKI使CML疾病相关死亡率下降，使总体生存（OS）显著改善，欧洲数据显示2/3的死亡患者并非与CML直接相关。ELTS（EUTOS Long-Term Survival）评分能更好识别伊马替尼一线治疗中相关死亡高风险患者，同样能够更好区分二代TKI一线治疗不同危险组间的长期生存[28]。2024年中国学者进一步提出一线服用伊马替尼或二代TKI患者发生治疗失败的临床预测模型[29]，结果显示该模型可预测伊马替尼和二代TKI治疗患者的累积治疗失败率，且区分能力优于Sokal和ELTS评分，适用于各个年龄阶段患者，不同风险组累积主要分子学反应（MMR）、分子学反应（MR）4.0、MR4.5发生率亦存在显著差异[29]。该模型显示出良好的预后预测能力，尤其适用于中国人群，但该模型作为传统评分系统（如ELTS）的补充，其远期预测价值及对治疗策略的指导意义，有待更多前瞻性研究数据进一步证实。本版指南推荐采用ELTS评分系统及来自中国患者数据的TKI治疗失败模型预测患者治疗结局（表1），并指导靶向药选择。

**表1:** 慢性髓细胞性白血病预后评分系统

评分系统	评分计算公式	低危	中危	高危
ELTS[30]	0.0025×（年龄/10）^3^+0.0615×脾脏大小+0.1052×原始细胞+0.4104×（PLT/1000）^−0.5^	<1.5680	1.5680～2.2185	>2.2185
TKI治疗失败预测模型[29]	0.1919×性别（男性=1，女性=0）+1.6160×（年龄/100）+0.3105×（血红蛋白浓度/100）^−2^+0.1087×外周血中原始细胞比例+0.0671×脾下缘超出范围的厘米数+0.5461×Ph^+^细胞中是否存在高风险额外染色体异常（是=1，否=0）	<1.3115	1.3115～2.4266	>2.4266

**注** PLT单位为×10^9^/L，年龄单位为岁，脾脏大小指肋缘下厘米数，原始细胞指外周血分类百分数。TKI：酪氨酸激酶抑制剂

所有数据应在CML治疗开始前获得，上述预后评分系统并非唯一确定的预后参数，越来越多研究显示遗传学及分子学异常与CML预后及TKI治疗反应相关，ACA与疾病分期相关（表1）。ACA根据其发生频率分为主要［+8，+Ph，i（17q），+19，+17，+21］或次要（−7/7q−，11q23，3q26.2）途径异常，ACA与复杂核型均被认为是高风险ACA，其预后取决于ACA的类型[23]。部分研究显示i（17q）、−7/7q−，或3q26.2异常伴随或不伴复杂核型为极高危ACA，与疾病快速进展相关[31]。因此无论Sokal和ELTS分层结果，伴ACA均为基线高危因素。

BCR::ABL1以外基因的体细胞突变在CML中不少见，尤其疾病进展时检出频率更高，ASXL1突变在约10％的患者中发现。部分研究表明，相关基因突变尤其是ASXL1突变与TKI反应较差和（或）无事件生存期缩短相关，但对OS期无显著影响[27],[32]–[35]。

二、治疗方案推荐

（一）治疗目标

随着伊马替尼为代表TKI的出现，CML患者长期结局发生革命性变化，CML已进入慢性病管理时代。靶向药治疗下绝大多数CML慢性期患者可以实现正常的预期寿命，且部分维持稳定DMR患者可达到TFR[22]。目前CML治疗目标包括：减少疾病进展使CML患者寿命正常化（长期生存）；在保证疗效的同时，应尽可能减少药物不良反应，改善患者生活质量；提高持久DMR比例，使更多患者有条件进行TFR尝试，达到功能性治愈。

（二）慢性期治疗

1.靶向治疗：目前在国内外已获批用于CML慢性期的靶向药包括两类：①传统的ATP竞争性TKI，包括一代伊马替尼，二代尼洛替尼、达沙替尼、博苏替尼、氟马替尼，三代泊那替尼、奥雷巴替尼；②STAMP变构药物阿思尼布。

（1）一线治疗：一线靶向药选择应当在明确治疗目标基础上，依据患者初诊预后分层、年龄、个体状况、基础疾病、合并用药以及靶向药物不良反应特征恰当选择[19]–[23],[36]。对于年轻患者，终身治疗带来的经济压力和长期不良反应，以及育龄期女性患者的生育需求，TFR与OS同样重要。对于老年患者尤其是伴有需要用药治疗合并症者，更注重长期治疗的安全性，OS应作为主要的目标，TFR成为额外获益。不同TKI器官毒性存在显著差异，应成为一线及后线治疗选择的重要考虑因素。

NMPA批准慢性期患者一线治疗的药物包括伊马替尼、尼洛替尼、氟马替尼、阿思尼布。在获得CCyR、早期分子学反应（Early molecular response, EMR）、MMR、DMR以及减少疾病进展方面，二代TKI相比伊马替尼均有优势，但接受伊马替尼一线治疗患者的长期生存与二代TKI相似，且未被二代TKI超越[13]。ASC4FIRST随机对照研究[18]显示阿思尼布一线治疗相较一代及二代TKI具有MMR、DMR优势，且更少的脱靶作用、不良反应导致停药的风险低，但长期数据仍有待观察。伊马替尼相关不良事件主要为体液潴留、肌肉痉挛及胃肠道毒性、肾功能损害，显著影响患者生活质量[37]。尼洛替尼心血管事件风险在60岁以上患者进一步增加[38]。达沙替尼治疗相关胸腔积液、肺动脉高压发生与剂量及患者年龄相关[16]。氟马替尼治疗相关腹泻和丙氨酸转氨酶升高以及肾脏损害值得关注[39]–[40]。ASC4FIRST研究中位随访16.3个月，伊马替尼组未报告动脉栓塞性事件（Arterial occlusive events），阿思尼布组为1％，二代TKI组为2％[18]。

参照NCCN、ELN指南，结合药物的可及性[13]–[18],[20]–[23],[36],[41]，本指南推荐的CML慢性期一线治疗药物详见图1：一代TKI：伊马替尼400 mg，每日1次；二代TKI（按首字母顺序排列）：达沙替尼100或50 mg，每日1次（NMPA未批准达沙替尼在CML一线应用）；氟马替尼600 mg，每日1次；尼洛替尼300 mg，每日2次；变构药物阿思尼布80 mg，每日1次。TKI治疗期间应定期监测血液学、细胞遗传学及分子学反应，定期评估患者治疗耐受性，参照里程碑反应（表2）进行治疗反应评估，调整后续治疗策略（图2）。

**图1:**
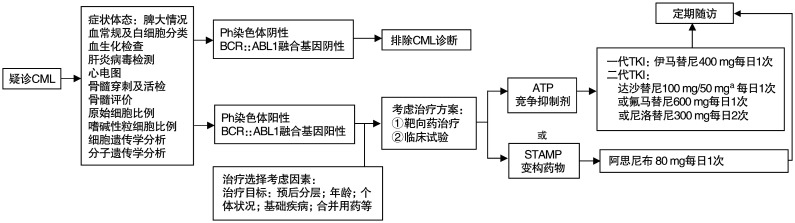
初诊慢性髓细胞性白血病（CML）慢性期的诊断及初始治疗流程 **注** 结合治疗目标以及选择考虑因素，对于追求无治疗缓解者，需要达到快而持久的深度分子学缓解，应选择疗效更强且安全性佳的药物，如二代酪氨酸激酶抑制剂（TKI）或阿思尼布；有合并症或耐受性较差的患者，需要选择安全性佳的药物，如伊马替尼或阿思尼布。^a^达沙替尼初始剂量按照说明书给予100 mg每日1次，基于达沙替尼剂量探索研究，起始剂量50 mg每日1次，可获得较好的安全性

**表2:** 慢性髓细胞性白血病慢性期的治疗反应里程碑

治疗反应	基线	3个月	6个月	12个月	任何时间
良好（无需转换治疗）		≤10％	≤1％	≤0.1％	≤0.1％
警告（可能需要转换治疗）	高危ACA，ELTS评分高危	>10％	>1％～10％	>0.1％～1％	>0.1％～1％ 丧失≤0.1％（MMR）
不佳（高耐药风险，推荐转换治疗）		若证实>10％	>10％	>1％，1％～10％^a^	丧失既往反应，耐药性激酶区突变，高危ACA

**注** ^a^主要基于BCR::ABL1结果评估治疗反应里程碑；判定需要个体化，不应仅根据单一结果更换靶向治疗，应观察反应趋势和患者的个体情况；如治疗目标是长期生存，在12个月时BCR::ABL1在>1％但<10％，是可被接受的；如治疗目标是追求无治疗缓解，在12个月时BCR::ABL1在>1％可考虑转换治疗。ACA：额外的染色体异常。MMR：主要分子学反应

**图2:**
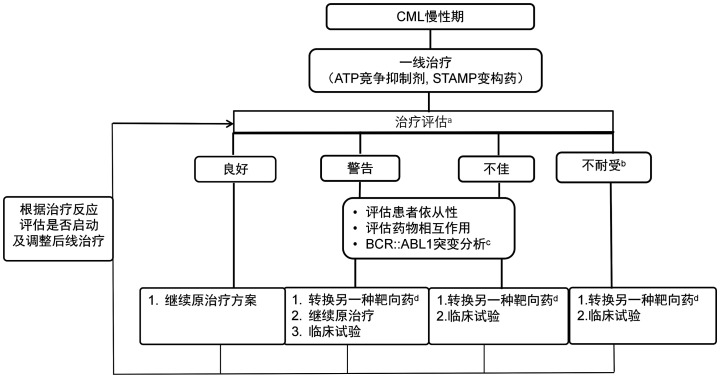
慢性髓细胞性白血病（CML）慢性期二线及以上治疗调整策略 **注** ^a^如果达到持续深度分子学反应（DMR），可以考虑尝试无治疗缓解（TFR），详见TFR章节。需注意停药后的疾病监测和管理非常重要，对既往治疗反应不佳或警告的患者，后期换用治疗后达到TFR的标准，可以尝试TFR，但需谨慎。^b^不耐受（剂量减低或中断仍无法耐受）：包括因不良反应或耐受性对药物做出调整，因严重或反复不良反应换用药物详见第八章节。^c^基于BCR::ABL1突变结果的治疗选择，参照表4。^d^既往对于某一靶向药耐药或者不耐受，不再考虑更换为该药物；对于治疗反应不佳需换药者，也不建议更换为伊马替尼

e13a3或e14a3转录本约占CML人群的0.36％，该转录本缺乏编码SH3结构域的ABL第二外显子[42]，导致阿思尼布耐药[43]。

一线药物治疗第3、6和12个月时国际标准化（IS）BCR::ABL1水平纳入里程碑反应，与2020年版中国指南基本保持一致，对治疗反应定义更新为良好（无需治疗更换）、警告（可能需要治疗更换）和不佳（首选治疗更换），同时对12个月反应不佳定义分层。

针对12个月时的分子学反应，MD Anderson数据显示TKI治疗2年BCR::ABL1^IS^<10％的患者10年生存率>90％，BCR::ABL1^IS^>10％的患者10年生存率为80％[44]。德国CMLIV研究[45]显示TKI治疗12个月时BCR::ABL1^IS^>1％患者的10年生存率约为80％，比12个月时BCR::ABL1^IS^≤0.1％的患者低10％，12个月时BCR::ABL1^IS^>10％的患者，其OS率下降至55％，且老年患者的生存率低于年轻患者，主要死于非CML相关疾病，提示有合并症的CML患者（通常为老年人）转换具有明显不良反应的药物可能会造成更为严重的伤害。因此，针对以OS为主要目标的人群，12个月时BCR::ABL1在>1％但<10％反应可被接受。12个月内实现良好里程碑反应与后期的深度分子反应相关，更高比例患者尝试TFR[46]–[47]。

靶向药治疗3个月BCR::ABL1^IS^>10％是否调整治疗一直存在争议，缺乏有力证据表明靶向药转换后结局更好。多数研究显示TKI治疗3个月BCR::ABL1^IS^>10％患者长期生存良好[44]–[45]。伊马替尼治疗3个月BCR::ABL1^IS^>10％患者更换为达沙替尼治疗可以提高MMR和MR4获得率，但无生存获益[48]。

CML管理应当个体化，不建议根据BCR::ABL1^IS^的单次、单一数值做出转换治疗的决策。强调在对治疗策略进行重大调整前，必须结合治疗目标、依从性、患者个体情况等解读疗效里程碑反应。未达到里程碑反应，但BCR::ABL1^IS^水平稳定下降的患者，治疗转换可能是非必须的。药物中断/剂量减少（尤其是靶向药治疗初期）、依从性不足等亦可能导致治疗反应不达标，对无明确耐药患者，应积极进行宣教，提高患者的治疗依从性，结合后续治疗反应进行药物调整。能够耐受更强效药物而无明显不良反应的年轻患者、健康状况良好无合并症的老年患者、追求TFR患者，未达预期里程碑反应者可进行ABL1突变分析，考虑靶向药转换。

对治疗随访过程中未能达到治疗里程碑反应的患者，推荐进行骨髓穿刺细胞形态学和细胞遗传学检测,明确疾病状态。

（2）二线及以上治疗：治疗反应未达预期、药物相关不良反应成为后线药物调整的主要原因。CML治疗过程出现无法耐受的不良反应，经过减量或者暂停用药仍无法缓解，可依照一线治疗原则选择后续治疗，换用其他获批的靶向药物。

里程碑反应未达预期患者，基于治疗目标，综合考虑患者病史、合并症、合并用药、药物不良反应、医师经验及药物可及性，并结合ABL激酶区突变类型评估是否需要更换治疗[20]–[23],[41]。①治疗警告：及时评估ABL激酶区突变，参照突变情况选择敏感药物；对无耐药性突变患者，在密切监测的同时，考虑治疗成本、风险及获益等，决定是否更换或者维持原治疗。②治疗反应不佳：参照ABL激酶区突变类型选择敏感药物，无明确可识别突变的患者，后续治疗应参考患者的年龄、治疗耐受性、合并症以及药物的不良反应等选择。

因治疗反应不佳/警告需要更换TKI者，二线可选更高代TKI或者阿思尼布，原则上选择作用更强、突变覆盖更广泛或者不同机制的TKI，或临床试验。

三线及三线后治疗患者更具异质性，多数存在二代TKI耐药，优先推荐三代TKI奥雷巴替尼、泊那替尼或变构药物阿思尼布或临床试验[23],[40]，临床应用中尚需考虑相关药物NPMA推荐适应证。

CML慢性期患者后线疗效数据可参考表3。若存在可识别的、导致靶向药治疗耐药的BCR::ABL1突变时，可参照表4进行敏感药物选择。体外研究提示氟马替尼对T315I、Y253F、E255K突变耐药[57]，但缺乏大规模、长期随访数据系统性评估氟马替尼治疗过程中的突变发生情况及敏感或耐药的突变情况。多数情况下ATP位点的耐药突变和STAMP位点的耐药突变不重叠[58]，故对ATP竞争抑制剂耐药可换用STAMP变构药物阿思尼布，对阿思尼布耐药也可换用ATP竞争抑制剂。

**表3:** 慢性髓细胞性白血病慢性期（CML-CP）二线以上治疗的长期随访结果（％）

靶向药	组别（例数）	中位随访时间	MCyR	CCyR	MMR	MR4.5	OS率	PFS率
DAS 100 mg每日1次[49]	IM耐药（124例）	7年	–	–	43	20	63	39
	IM不耐受（43例）		–	–	55		70	51
Flu 600 mg每日1次[40]	IM、DAS或NIL耐药或不耐受（199例）	11.4个月	–	61.2	56.4	–	–	–
NIL 400 mg每日2次[50]	IM耐药（226例） IM不耐受（95例）	4年	59	45	–	–	78	57
Pona 45 mg每日1次[51]	NIL或DAS耐药或不耐受（203例）	57个月	56	49	35	–	76（5年）	52（5年）
	CML-CP伴T315I突变（64例）	57个月	72	70	58	38	66（5年）	50（5年）
奥雷巴替尼40 mg隔日1次[52]–[53]	IM、DAS和NIL一种及以上耐药或不耐受（127例）^a^	34.3个月	79	69	56	39	94（3年）	92（3年）
	Pona耐药或不耐受（30例）	48周	–	57.7	36.7	–	–	–
	阿思尼布耐药或不耐受（17例）	48周	–	60	29.4	–	–	–
	CML-CP伴T315I突变（77例）^b^	34.3个月	85.3	76	68.7	54.7	–	–
阿思尼布80 mg每日1次[54]	IM、DAS、NIL、BOS其中一种耐药或不耐受（71例）	48周	–	84	68.3	–	–	–
阿思尼布40 mg每日2次[55]	IM、DAS、NIL、Pona、Rado两种及以上耐药或不耐受（157例）	2.3年	–	39.8	37.6	10.8	94（2年）	97（2年）
阿思尼布200 mg每日2次[56]	CML-CP伴T315I突变（45例）	2年	–	62.2	48.9	24.4	–	–

**注** MCyR：主要细胞遗传学反应；CCyR：完全细胞遗传学反应；MMR：主要分子学反应；MR4.5：分子学反应4.5；OS：总生存；PFS：无进展生存；IM：伊马替尼；DAS：达沙替尼；NIL：尼洛替尼；Flu：氟马替尼；Pona：泊那替尼；BOS：博苏替尼；Rado：拉多替尼；–：无资料。a：包含b中77例伴T315I突变的患者

**表4:** 不同靶向药物耐药性突变[20]

治疗药物	禁忌用药的耐药性突变
氟马替尼	T315I、Y253H/F、E255K/V
达沙替尼	T315I/A、F317L/V/I/C、V299L
尼洛替尼	T315I、Y253H/F、E255K/V、F359V/C/I
泊那替尼	无
奥雷巴替尼	无
阿思尼布	A337V/T、L340Q、A344P、A433D、G463D/S、P465S/Q、V468F、F497L、I502L/N、V506L/M、M244V、F359V/I/C，E13A3或E14A3转录本

**注** 特异性突变体对各种酪氨酸激酶抑制剂的相对敏感性研究主要通过体外模型进行，可能与体内情况存在差异。氟马替尼耐药谱系基于临床前研究，临床耐药突变谱需更多临床数据证实。阿思尼布的突变集中在肉豆蔻酰口袋附近，与ATP位点的突变不重合，出现耐药突变后可考虑更换ATP竞争抑制剂。复合、复杂突变应优先考虑奥雷巴替尼、泊那替尼或异基因造血干细胞移植

对于无明确可识别突变的患者，后续靶向药治疗的选择应基于患者的年龄、治疗耐受性、合并症以及药物的不良反应来选择。

（3）其他治疗：因各种原因无法使用靶向药物治疗的患者可考虑以下治疗方案。

①干扰素为基础的方案[37]：随着TKI选择多样化，药物价格更为经济，干扰素基本退出CML治疗。但对多种TKI治疗失败、不耐受且无法移植、妊娠期患者仍可考虑干扰素为基础的治疗。

②allo-HSCT：在TKI治疗时代，结合药物可及性等综合因素考虑，allo-HSCT作为多种靶向药治疗反应不佳或不耐受的晚期治疗选择，应当严格掌握适应证。详见后文。

（三）进展期治疗

进展期患者推荐参与临床试验和（或）接受allo-HSCT。

初始进展患者比慢性期治疗后进展的患者预后更好。初始加速期患者按照慢性期高危治疗并随访，结合治疗反应评估allo-HSCT的必要性及可行性。TKI治疗进展为加速期的患者参照ABL激酶区突变选择TKI，优先选择三代TKI，并桥接allo-HSCT[20],[23],[59]。

无论初始急变还是慢性期/加速期后进展为急变的患者，建议在敏感TKI治疗的基础上（推荐优先选择三代TKI），依据急变类型联合化疗（含低强度化疗），恢复至慢性期后尽快桥接allo-HSCT。对于不适合allo-HSCT患者，推荐采用以TKI为基础的维持治疗。

三、TKI治疗反应定义以及治疗反应的监测

CML患者接受TKI治疗过程中疗效评价包括血液学、细胞遗传学以及分子学反应，及时评价治疗反应对于优化治疗具有重要且积极的意义。CML慢性期患者的血液学、细胞遗传学以及分子学反应的标准、监测评估方式以及频率参考2020年版中国CML诊断与治疗指南[41]。分子学监测仍为主要的监测方式，但对未达良好疗效、反复发生严重血液学毒性的患者应同时进行细胞遗传学检测。

里程碑反应警告或不佳、疾病进展的患者推荐BCR::ABL1突变检测。Sanger测序一直是BCR::ABL1突变分析的金标准，但灵敏度较低，难以检测到<15％～20％的突变克隆。靶向二代测序（NGS）敏感性更高，能够进行克隆分析以及复合突变鉴定，有条件地区可采用经过验证并基于cDNA的NGS检测进行BCR::ABL1突变筛查。亦可采用灵敏的数字PCR（ddPCR）检测常见突变[23]。

四、TFR

1. TFR可行性：目前全球范围进行了多项TKI停药试验，每项试验的入选标准略有不同，重启TKI治疗标准有所不同，但结果非常相似：40％～50％的患者可维持持续TFR[60]–[68]。

2. TFR停药标准：持续MR4.0/MR4.5以上分子学反应超过2年是目前停药试验的前提条件。TKI服药时间、反应深度及停药前DMR持续时间与成功停药呈正相关[60]–[68]。

3. TFR失败并启动再治疗标准：以丧失MMR为标准，临床操作安全、可行[62]–[66]。

4. TFR失败再治疗效果：TFR失败患者，多数对停药前TKI再治疗敏感，86％～100％患者能够再次获得MMR，80％以上患者再次获得DMR[60]–[66]。

5. TFR患者筛选[23]：在满足必备条件基础上，参考最低和最佳标准，结合患者具体情况择机进行停药尝试。必备条件：

（1）首次CML慢性期患者（其他情况缺乏数据支持）。

（2）患者有停药意愿且沟通良好。

（3）可靠的BCR::ABL1定量检测方法，灵敏度至少达到MR4.5，且结果反馈迅速。

（4）若为非典型转录本，需在具备高标准定量检测能力的实验室进行监测。

（5）患者同意停药后接受更频繁的监测。

最低标准：

（1）一线治疗患者；或因不耐受、对TKI敏感的突变换用二线药物患者。

（2）典型的e13a2或e14a2型BCR::ABL1转录本。

（3）若为非典型转录本，需在具备高标准定量检测能力的实验室进行监测。

（4）TKI治疗持续时间>5年（二代TKI需>4年）。

（5）深度分子学反应（DMR，即MR4.0或更好）持续时间>2年。

最佳标准：

（1）TKI治疗持续时间>5年。

（2）若为MR4.0，DMR持续时间>3年。

（3）若为MR4.5，DMR持续时间>2年。

6. 停药后随访监测：部分患者出现TKI停药综合征，表现为停药后肌肉骨骼疼痛和（或）瘙痒出现或加重，持续数月后可缓解,部分患者需要服用止痛药物对症治疗缓解症状。

多数停药复发发生于停药后6个月内，部分患者晚期复发。停药6个月内、6～24个月和24个月后复发患者占比分别为69％、14％和17％[68]–[69]。尽管TFR后疾病进展至急变期极为罕见（见下文），但其后果严重，且无法预测。因此停药后需要终身规律的监测，及时发现复发，丧失MMR患者需立即重启TKI治疗。停药后监测如下：

（1）停药后：前6个月：每1～2个月进行1次分子学监测。第7～12个月：每2个月监测1次。之后（无限期）：对于持续保持MMR的患者，每3个月监测1次。

（2）若患者失去MMR，应尽快恢复TKI治疗，每月进行分子学监测，直至再次获得MMR；之后每3个月监测1次。重新启动TKI治疗后3个月内未恢复MMR，应进行BCR::ABL1激酶结构域突变检测。

7. 停药后疾病进展：TFR后疾病进展罕见且无法预期。文献报道TFR期间急变多数在TKI停药后至少12个月发生，最长间隔为67个月，且TFR急变的发生率低于0.1％[70]–[71]。需要注意部分真实世界中停药后急变未报道，可能低估了TFR后急变情况。

五、靶向药的剂量优化

尽管CML患者的结局已得到改善，但在提高TFR、减少长期不良事件、改善生活质量等方面仍有待提升。患者管理重点包括预防和（或）减轻不良反应，提高生活质量。在结合疗效和安全性基础上进行主动性剂量调整，而非仅仅是不良事件驱动的被动降低剂量成为靶向药治疗最佳管理一部分。

1. 低剂量初始治疗：目前对于起始剂量的优化的前瞻性研究数据不足，仅有少量回顾性研究，因此TKI的起始剂量建议使用标准剂量。对于年轻患者、能耐受标准剂量TKI、肿瘤高负荷、强烈希望停药及高危CML慢性期的患者，开始使用标准剂量可能是最佳策略。对部分TKI初始剂量探索的研究主要包括达沙替尼、泊那替尼和奥雷巴替尼。

（1）达沙替尼：对于CML慢性期患者推荐达沙替尼起始剂量每日100 mg。对于低危或中危CML慢性期患者，一线达沙替尼每日50 mg与每日100 mg具有相似的疗效[72]。间歇性给药或剂量减少至每日50 mg对于伊马替尼耐药/不耐受的CML慢性期患者二线及后续治疗有效[73]–[76]。因此，对于临床上每日100 mg达沙替尼有显著不耐受的患者，应考虑使用50 mg（老年患者可在密切监测下使用20 mg），以避免发生导致达沙替尼停药的严重不良事件。但低剂量达沙替尼目前缺乏前瞻性随机研究数据，在ELTS高危患者中的数据有限，需权衡获益和风险调整剂量。

（2）泊那替尼：泊那替尼的推荐起始剂量每日45 mg，前瞻性OPTIC试验数据支持对于伴有T315I突变的患者每日45 mg泊那替尼开始治疗，达到BCR::ABL1^IS^≤1％后减少至每日15 mg维持[77]。回顾性研究显示每日15 mg具有较低的不良事件发生率，且对疗效无影响[78]。

（3）奥雷巴替尼：奥雷巴替尼推荐起始剂量40mg隔日1次，中国多中心回顾性研究在对其他TKI耐药CML慢性期患者中对比30 mg和40 mg起始治疗的疗效和安全性[79]。采用倾向性评分匹配矫正两组不平衡的基线特征，两组细胞遗传学反应、分子学反应及生存结果相当，且30 mg隔日1次的耐受性更好，持续治疗比例更高。

2. 获得疗效后TKI剂量降低或间歇给药：部分研究结果显示，无既往TKI耐药、接受TKI治疗≥2年且维持MMR及以上反应患者，TKI剂量递减安全可行[80]–[82]。

六、allo-HSCT在CML中的应用

TKI问世后，CML患者接受allo-HSCT的比例大幅下降，allo-HSCT不再是CML慢性期患者的一线治疗选择，但依然是CML根治性手段，尤其对TKI耐药以及进展期患者。推荐考虑allo-HSCT人群包括：①≥三代TKI和变构药物阿思尼布治疗失败的慢性期患者；②对多种TKI不耐受的慢性期患者；③TKI治疗期间疾病进展的患者；④初始加速期但对一线TKI治疗的应答丧失且无可替代敏感药物的患者；⑤急变期的患者；⑥TKI治疗下，Ph染色体阴性但出现−5/5q−、−7/7q−异常的患者。

近期欧洲多中心904例CML患者移植的数据显示，首次慢性期、KPS评分>80分具有更高的移植后OS率和无进展生存率[83]。既往大量研究显示处于第二次慢性期的患者移植后的生存结局明显优于CML加速期或CML急变期患者[20]。因此，进展期患者回到慢性期后应尽快移植，急变的高危人群避免“错过”慢性期窗口期。

既往研究显示allo-HSCT后6～12个月转录本持续阳性的患者复发率远高于移植后18个月检测阳性的患者，因此移植后应常规进行残留病的监测。allo-HSCT后疾病评价包括血液学、骨髓细胞及染色体核型分析或者FISH、分子学分析。①达到CCyR且分子学检测结果为阴性患者：进行骨髓和（或）外周血PCR监测，每3个月1次，连续2年，随后每6个月1次，连续3年。②获得CCyR但分子学检测阳性患者：检测是否存在ABL激酶突变，挑选靶向药物治疗；或调整免疫抑制剂、供者淋巴细胞输注、干扰素、高三尖杉酯碱等治疗；或参加临床试验。③未获得CCyR或复发的患者：停止免疫抑制治疗并监测，按照前述分子学监测阳性患者处理；或考虑二次移植。

CML患者移植后TKI预防性治疗减少复发，但预防治疗时长无确定结论[23]，通常至少2年。

七、靶向药治疗期间的妊娠管理

动物实验数据显示，伊马替尼、氟马替尼、尼洛替尼、达沙替尼、奥雷巴替尼、泊那替尼和阿思尼布具有生殖毒性，而无遗传毒性。现有的临床资料显示，TKI治疗可能一过性影响雄性激素，但对男性患者生育能力无显著影响，男性CML患者服用TKI期间配偶受孕所生子女无明显增加先天畸形的风险，女性配偶的流产率亦无明显增加。女性患者妊娠情况较复杂，妊娠期间服用TKI增加流产率和致畸风险。女性患者服用TKI期间意外妊娠者绝大部分在发现怀孕后停药，多数妊娠结果良好，但大宗病例的报道显示，有近10％的畸胎率[84]–[87]。伊马替尼可以通过血胎盘屏障，并分泌入乳汁；尼洛替尼较少通过血胎盘屏障，也可分泌入乳汁。羟基脲等细胞毒性药物具有潜在的致畸作用。分子量较大、不易透过血胎盘屏障的干扰素α已被较多文献确认为各期妊娠患者的安全选择[88]。

1. 计划妊娠：

（1）男性患者一般建议无需停用TKI治疗，但相关经验有限。也可在开始TKI治疗前进行精子冷冻保存，但目前缺乏未经治疗的CML患者精子质量的数据。

（2）女性患者不建议在TKI治疗期间计划妊娠，育龄期女性开始TKI治疗前可考虑进行卵子冻存。由于流产率增高和胎儿畸形的可能，女性在备孕及妊娠期间应停止TKI的治疗。因此女性患者未获MMR者应避免计划妊娠。分娩后可恢复TKI治疗，TKI治疗期间避免哺乳。考虑到药物对胎儿器官形成的影响，结合部分意外妊娠结局数据，女性患者可持续使用TKI直至首次妊娠试验阳性（距末次月经约4周，即妊娠4周）后停用TKI，最大限度地减少关键孕早期胎儿的TKI暴露[89]。

2. 意外妊娠及妊娠期间疾病的监测[20],[23]：由于致畸风险，应避免在妊娠期（尤其是妊娠前3个月）使用TKI治疗。

（1）妊娠期间确诊CML的患者：处于加速期或急变期的患者，建议立即终止妊娠，并立即开始TKI和（或）化疗。对于慢性期的患者，尽可能避免应用TKI、羟基脲和白消安等具有致畸可能的药物，可定期采用血细胞分离术和（或）干扰素维持血液学相对稳定；当白细胞分离术不能满意地控制血小板计数时，可予以阿司匹林或低分子肝素抗凝；若上述方法不耐受或不能满意控制血细胞，妊娠16周后可短期启用伊马替尼400 mg/d，伊马替尼耐药或不耐受可考虑尼洛替尼400～600 mg/d。

（2）TKI治疗中女性患者妊娠的处理：在充分知情下，选择保留胎儿应立即中断TKI治疗，严密监测疾病状况，治疗参考上述原则。产后依照疾病状态明确何时重启TKI治疗。TKI再治疗后避免哺乳。

八、靶向药物不良反应的管理

绝大多数患者需要长期服用TKI，患者管理的很大一部分重点在于预防和（或）减轻毒性反应，同时提高生活质量。不良事件可能由既往合并症、与其他药物的相互作用，或单纯的衰老过程引起。治疗中应参照药物说明书、治疗反应、2020年版中国CML诊断与治疗指南的不良反应处理章节[41]进行药物调整及对症支持治疗。

所有靶向药物都可能引起血液学不良反应，如中性粒细胞减少症、血小板减少症和贫血，30％～80％的患者会受到影响，在治疗的前2个月或血细胞计数恢复正常之前，需要每1～2周监测1次全血细胞计数。减量或者暂停TKI通常可缓解血液学不良反应。如果反复出现和（或）长时间的3～4级毒性，减量后仍无法耐受应考虑换用其他靶向药物治疗。

不同靶向药物的非血液学不良反应特征用于指导初始及后线治疗选择。建议更换药物的相对特异性不良反应包括：剂量减少后仍复发胸腔积液或肺动脉高压（常见于达沙替尼）；复发性或严重胰腺炎（尼洛替尼相对多见，泊那替尼、阿思尼布及其他药物临床研究中亦有脂肪酶增高）；心血管事件（泊那替尼、奥雷巴替尼和尼洛替尼更常见）；器官免疫介导的炎症（肺炎、心肌炎、肾炎，目前靶向药均可能导致）。伊马替尼与肾脏不良事件相关，换用达沙替尼或尼洛替尼可显著改善肾功能。达沙替尼相关的肾脏事件多为肾病综合征，换用伊马替尼可缓解[90]。氟马替尼治疗后部分患者出现肌酐水平增高，应密切监测及时调整治疗策略，避免肾功能损伤加重[39]–[40]。出现以上非血液学不良事件，进行监测并积极支持性干预，如在减量和暂停用药后仍无法解决，可考虑更换另一种靶向药物，但注意避开既往已经使用过但耐药或者不耐受的靶向药物。
